# Immune Effector Recovery in Chronic Myeloid Leukemia and Treatment-Free Remission

**DOI:** 10.3389/fimmu.2017.00469

**Published:** 2017-04-24

**Authors:** Amy Hughes, Agnes S. M. Yong

**Affiliations:** ^1^Department of Haematology, SA Pathology, Adelaide, SA, Australia; ^2^Cancer Theme, South Australia Health and Medical Research Institute (SAHMRI), Adelaide, SA, Australia; ^3^School of Medicine, The University of Adelaide, Adelaide, SA, Australia

**Keywords:** immunology, chronic myeloid leukemia, treatment-free remission, immune surveillance, deep molecular response

## Abstract

Chronic myeloid leukemia (CML) is a hematological cancer, characterized by a reciprocal chromosomal translocation between chromosomes 9 and 22 [t(9;22)], producing the Bcr-Abl oncogene. Tyrosine kinase inhibitors (TKIs) represent the standard of care for CML patients and exert a dual mode of action: direct oncokinase inhibition and restoration of effector-mediated immune surveillance, which is rendered dysfunctional in CML patients at diagnosis, prior to TKI therapy. TKIs such as imatinib, and more potent second-generation nilotinib and dasatinib induce a high rate of deep molecular response (DMR, *BCR-ABL1* ≤ 0.01%) in CML patients. As a result, the more recent goal of therapy in CML treatment is to induce a durable DMR as a prelude to successful treatment-free remission (TFR), which occurs in approximately half of all CML patients who cease TKI therapy. The lack of overt relapse in such patients has been attributed to immunological control of CML. In this review, we discuss an immunological timeline to successful TFR, focusing on the immunology of CML during TKI treatment; an initial period of immune suppression, limiting antitumor immune effector responses in newly diagnosed CML patients, linked to an expansion of immature myeloid-derived suppressor cells and regulatory T cells and aberrant expression of immune checkpoint signaling pathways, including programmed death-1/programmed death ligand-1. Commencement of TKI treatment is associated with immune system re-activation and restoration of effector-mediated [natural killer (NK) cell and T cell] immune surveillance in CML patients, albeit with differing frequencies in concert with differing levels of molecular response achieved on TKI. DMR is associated with maximal restoration of immune recovery in CML patients on TKI. Current data suggest a net balance between both the effector and suppressor arms of the immune system, at a minimum involving mature, cytotoxic CD56^dim^ NK cells may be important in mediating TFR success. However, a major goal remains in CML to identify the most effective pathways to target to maximize an advantageous immune response and promote TFR success.

## Introduction

Chronic myeloid leukemia (CML) is a hematological cancer characterized by the presence of the *BCR-ABL1* oncogene, which is the product of a reciprocal translocation between chromosomes 9 and 22 [t(9;22)], in a hematopoietic stem cell. The resultant constitutively active tyrosine kinase, Bcr-Abl, mediates phosphorylation and activation of downstream signaling pathways, causing altered cell adhesion, inhibition of apoptosis, differentiation arrest, and proteasomal degradation of key proteins, which lead to the phenotype of the disease (1). CML is considered to be one of the cancers most sensitive to immunological manipulation (2). Tyrosine kinase inhibitors (TKIs) imatinib, nilotinib, and dasatinib are used as first-line treatment in CML. These small molecule inhibitors block the adenosine triphosphate-binding site of the Bcr-Abl tyrosine kinase and prevent phosphorylation of downstream effector proteins. Clinical response to treatment is assessed initially by monitoring the reduction of the peripheral white blood cell count, and subsequently by measurement of *BCR-ABL1* transcript levels against a control gene (3). An optimal response following initiation of TKI treatment is a major goal, as this confers improved patient survival. Clinical guidelines on optimal molecular responses refer to achievement of target *BCR-ABL1* levels [e.g., ≤0.1%, major molecular response (MMR)] at specific timepoints (4). The more recent goal in CML treatment is to induce a durable deep molecular response (DMR; *BCR-ABL1* ≤ 0.01%) as a prelude to successful treatment-free remission (TFR), which occurs in approximately half of all CML patients who cease TKI therapy (5, 6). The lack of overt relapse in such patients has been attributed to immunological control of CML (7), although its precise mechanisms are as yet unclear.

Suppression of the innate and adaptive immune system, linked to an accumulation of immature myeloid cells (myeloid-derived suppressor cells, MDSCs), predominates during pathological conditions such as cancer, and leads to inhibition of the host–antitumor immunity (8). MDSC expansion in the blood and bone marrow at the time of diagnosis has been shown in patients with multiple myeloma (9), chronic lymphocytic leukemia (10), and acute myeloid leukemia (11). In CML, increased levels of MDSC, which originate from the malignant *BCR-ABL1* clone, are also observed, and these MDSC subsequently reduce following highly efficacious TKI therapy (12, 13). MDSCs promote the recruitment and expansion of other suppressor cells (regulatory T cells, Treg), leading to impaired innate effector natural killer (NK) cells and inhibition of T cell proliferation and activation, further downregulating antitumor immune surveillance that subsequently influence leukemia development and progression (14). In support, quantitative and functional defects of NK cells and diminished cytotoxic T lymphocyte (CTL) function have also been described in chronic phase (CP) CML patients at diagnosis (12, 15–17). Thus, the changing ratio between resident immune effector and immune suppressor cells in untreated CML and other hematological cancers, limits the patient’s immune status such that a predominantly immune inhibitory leukemic milieu is present, accounting for a diminished anti-leukemic effector immune response to control leukemia progression and/or relapse. Very recently, an increased proportion of mature, adaptive-like CD56^dim^ NK cells have been observed in CML patients who successfully discontinued imatinib (18). Other immunologic mediators such as plasmacytoid dendritic cells (pDCs), which may serve as promising prognostic factors for successful TFR, are also currently under investigation (19). TKIs also exert significant off-target multikinase inhibitory effects, albeit with differing potencies. Cumulative data suggest that TKIs exhibit a dual mode of action; direct oncokinase inhibition interspersed with concomitant immunomodulatory effects, particularly against key suppressor MDSC and Treg populations, conferring immune system re-activation and restoring effector-mediated immune surveillance (2, 13, 20–24). In this review, we discuss an immunological timeline to successful TFR in CML; an initial period of immune dysfunction in newly diagnosed CML patients, followed by restoration of immune effector responses and release of immune suppressors, albeit with differing frequencies in concert with differing levels of molecular response achieved on TKI. Optimum restoration of endogenous immune surveillance mechanisms may promote sustained TFR following TKI discontinuation attempt.

## Immune Dysfunction in Newly Diagnosed CML Patients

The majority (~90%) of CML patients are diagnosed while in CP, characterized by an expansion of circulating myeloid cells, which are mainly mature, and maintained by a small subset of disease initiating leukemic stem cells (LSCs) (25). Persistent immune dysfunction in CML patients at the time of diagnosis, prior to the start of any therapy is well documented, precluding the development of adequate anti-leukemia immune responses and promoting disease progression in the absence of highly efficacious TKI therapy. An essential role of the immune system, in particular that of innate and adaptive immune cells (i.e., NK cells, CD8^+^/CD4^+^ T cells), effector molecules, and endogenous signaling pathways, is to confer host protection against cancer (26). However, many tumors facilitate their self preservation and progression by the recruitment of immunosuppressive cells, release of inhibitory factors including immunosuppressive and inflammatory cytokines and upregulation of immune checkpoint pathways, in particular cytotoxic T-lymphocyte-associated protein 4 and programmed death-1 (PD-1) pathways (27, 28). The ligand for PD-1, programmed death ligand-1 (PD-L1), induces a coinhibitory signal in activated T cells and promotes T cell apoptosis, anergy, and functional exhaustion (29). Further research into better understanding this altered immune balance in CML patients at diagnosis is essential for the development of new therapeutic methods, aiming to augment antitumor immune activity and enhance TFR success rates following TKI cessation.

## Effector Cells of the Immune System in CML Patients at Diagnosis

The main antitumor effector cells of the immune system, NK cells, dendritic cells (DCs), and CTLs (30), play a direct role in host control of hematological malignancies, including CML. Antibody-secreting effector B cells, also called plasma cells also defend the body in an immune response, with distinct B cell subsets mediating different types of antibody responses (31). de Lavallade et al. (32) have previously reported loss of memory B cell subsets in CML patients at diagnosis; however, the clinical impact remains unclear, with no patients showing recurrent infections despite the B cell deficiency. In support, we have performed extensive characterization of major B cell subsets including transitional, naïve, non-switched memory, class-switched memory, plasmablasts, and plasma cells and observed non-switched memory B cell expression was decreased in CML patients at diagnosis compared to normal healthy donor samples (12).

Natural killer cells are lymphocytes and a critical component of the innate immune system, providing a front line defense against tumor cells. NK cells exert potent cellular cytotoxicity against malignant cells (CD56^dim^ NK cell subset) and produce immunoregulatory cytokines and chemokines, such as interferon-γ (IFN-γ) and tumor necrosis factor α (TNF-α), supporting the development of adaptive immunity (CD56^bright^ NK cell subset). In contrast to T cells that require interaction with professional antigen-presenting cells, such as mature DCs for activation, NK cells utilize a diverse array of inhibitory and activating receptors for target cell recognition and lysis with no requirement for prior antigen stimulation or clonal expansion (33). NK cells are dysfunctional in CP CML patients at diagnosis and NK cell numbers among lymphocytes are reduced, worsening with disease progression to advanced and blast crisis phase CML (15, 16, 34). The activating C-type lectin receptor NKG2D stimulates the cytotoxicity of NK cells following recognition of the stress-induced ligand major histocompatibility complex class I chain-related A (MICA). *BCR-ABL1* promotes DC-mediated NK cell activation by increasing the expression of NKG2D ligands including MICA (35). MICA releases soluble proteins produced on the surface of tumor cells, inducing negative modulation of NKG2D and facilitating tumor cell escape from NK cell killing (36–38). Reduced NKG2D-activating receptor expression has been previously identified in CML patients at diagnosis, promoting leukemic cell survival (36). Very recently, we identified reduced expression of the NKG2 family of C-type lectin receptors (CD94/NKG2A, CD94/NKG2C, and NKG2D) in CML patients at diagnosis (12). Similarly, the natural cytotoxicity receptors NKp30 and NKp46, the latter characterized as the major triggering receptor involved in NK cell cytotoxicity (39) and the killer immunoglobulin-like receptors (KIRs) KIR2DL2/DL3/DS2 were downregulated in CML patients at diagnosis compared to healthy donors. Downregulation of NKp30 and NKp46 has been reported previously in acute myeloid leukemia and chronic lymphocytic leukemia and shown to correlate with decreased NK cell cytotoxicity (40–42).

Dendritic cells are the most potent antigen-presenting cells in promoting activation of naïve T cells and play a critical role in the initiation and regulation of the immune response (43). DCs take up, process, and present antigens on major histocompatibility (MHC) class I and II molecules on the cell surface, to T cells, thus initiating antigen-specific immune responses and/or immunological tolerance (44). In normal peripheral blood, myeloid and pDC subsets are typically identified in the immature state in low numbers, in contrast, quantitative and functional defects including inefficient antigen presentation have been reported in DC subsets from CML patients (45–48).

Leukemia-associated antigen (LAA)-specific CTLs have been detected in the peripheral blood of CP CML patients, including CTLs specific for Bcr-abl and selectively expressed or overexpressed LAAs such as proteinase-3 (PR3) and Wilms’ tumor antigen 1 (WT1), and may be involved in the immunological control of CML (49–51). However, studies have shown T cells from untreated patients with CML are functionally impaired, displaying decreased TCRζ-chain expression, limited cytotoxic activity, and they do not produce immunoregulatory cytokines IFN-γ or TNF-α (52–54). TCRζ-chain expression is critical for normal T cell function, including proliferation and IFN-γ production (55). Molldrem et al. (56) suggest a novel escape mechanism from tumor immunity by leukemia-induced selective deletion of high avidity effector CTLs that have the greatest potency against CML.

CD62L downregulation may impair effector CTL immune responses in CML patients at diagnosis and abrogate anti-leukemic immune control, as CD62L is critical in controlling the traffic of T cells to secondary lymphoid tissues and priming by antigen-presenting cells. Sopper et al. (57) have reported decreased CD62L surface expression on T cells in CML patients at diagnosis. Another mechanism of impaired immune response in CML may involve aberrant PD-1/PD-L1 signaling on effector cells, and immunosuppressive Treg, which also express PD-1. PD-1 is temporarily expressed on activated immune effector cells under normal physiological conditions; however, constitutive expression results in diminished effector T cell function, and expansion of Treg with enhanced suppressor function, the latter playing a critical role in maintenance of the immunosuppressive tumor milieu (17, 58, 59). Mumprecht et al. (60) have previously reported PD-1 is upregulated on CML-specific CTLs and also on CTLs specific for unrelated antigens. Higher PD-1 expression in CTLs is related to inhibition of the effector phase of T cell responses and reduced T cell-mediated antitumor immunity (28). In addition, we have shown increased PD-1 expression on CD4^+^ and CD8^+^ T cells in CML patients at diagnosis, suggesting the presence of a broadly compromised immune system in CML patients at diagnosis (12).

## Suppressor Cells of the Immune System in CML Patients at Diagnosis

Myeloid-derived suppressor cells are a heterogeneous population of immature granulocytic and monocytic cells with potent immune suppressive functions and therefore represent one of the main suppressor cell populations within the immune system (61). MDSCs expand during cancer, inflammation, and infection and are characterized by the ability to suppress the cytotoxic function of T cells, including leukemia-specific CTLs, and NK cells (62). Cancer-derived inflammation drives the expansion and suppressive activity of MDSC, this is facilitated by multiple pro-inflammatory factors including GM-CSF and VEGF, produced within the immunosuppressive tumor microenvironment (63, 64). MDSC suppressive activity is mediated *via* a number of mechanisms, including increased production of reactive oxygen and nitrogen species and upregulation of arginase-1, the latter culminating in local depletion of arginine, an essential amino acid for T cell function (62, 65, 66) and depletion of cysteine, required by antigen-presenting cells including DCs, for effective T cell activation (67). Arginase-1 has also been shown to inhibit NK cell proliferation and secretion of IFN-γ (68) and MDSC induce anergy of NK cells through membrane-bound TGF-β1 (69). CML patients express high levels of monocytic and granulocytic MDSC at diagnosis compared to healthy donors (12, 13, 21), and these MDSC express *BCR-ABL1* and are part of the leukemia clone (13). In addition, CML serum leads to anergy of T cells, probably by increased arginase-1 expression (13) and coincubation of isolated monocytic and granulocytic MDSC of CML patients at diagnosis and autologous CFSE-labeled T cells has been shown to inhibit T cell proliferation, whereas control healthy donor MDSC displayed no suppressive activity (70).

Myeloid-derived suppressor cells also mediate the recruitment and expansion of immunosuppressive Treg, a specialized type of CD4^+^ T cell expressing the transcription factor forkhead box P3 (FoxP3), that can compromise the function of antitumor effector CD4^+^/CD8^+^ T cells and antigen-presenting cell activity to facilitate tumor cell immune evasion (62, 71–77). Zahran et al. (78) have reported the percentages of Tregs are significantly increased in newly diagnosed CML patients compared to controls, with lower Treg numbers in CP CML patients compared to accelerated and blast phases. In support, Bachy et al. (79) have shown that Treg are significantly increased in CML patients with intermediate or high-risk Sokal scores compared to low-risk patients. Thus, in a setting of high leukemic cell load in CP CML patients at diagnosis, CML cells can evade host immune surveillance by activation of the immune checkpoint receptor PD-1 and *via* PD-L1 upregulation, this signaling pathway influences immune suppression and disease progression, further supported by the recruitment of immunosuppressive MDSC and Treg cell populations (28).

## Immunogenicity of CML

The constitutively active Bcr-Abl kinase, while only weakly immunogenic in itself, leads to the upregulation of multiple genes, which may result in the expression of LAAs critical for priming of a protective antitumor CTL response against CML cells (80). The cure of CML following allogeneic hematopoietic stem cell transplantation (SCT) is attributed to the immunological graft-versus-leukemia effect, which is mediated by donor-derived T cells and NK cells, targeting alloantigens, primarily minor histocompatibility antigens (mHAgs) (81) and likely also LAAs (50, 82–84) and considered to play a critical role in disease eradication. Donor lymphocyte infusion after SCT provided the first direct evidence of the graft-versus-leukemia effect, in which, CTLs with mHAg specificity could salvage disease relapse (85, 86). The presence of LAA-specific CTLs directed against PR3 was associated with clinical responses to SCT (50, 84). In earlier functional studies, LAA-specific CTL responses to WT1 and PR3 were identified in CML patients and healthy donors, albeit at a lower frequency in the latter, and shown to expand in the recipient after transplantation, contributing to remission (83). Direct evidence of high *in vivo* antigen-specific immunogenicity against the LAA BMI-1 has also been found in the setting of SCT in CML (82).

Specific immunotherapies for CML patients targeting LAAs in combination with imatinib or other TKIs may exert a synergistic effect, inducing DMR in a high percentage of patients, while having the potential to overcome disease resistance by eliminating the quiescent CML stem cell subpopulation and enhancing specific immune responses against CML (80). In addition to BMI-1, WT1, and PR3, several other LAAs have been identified in CML patients such as RHAMM/CD186 and PRAME (82, 87–89) and thus represent candidate antigens for specific immunotherapies, with efficacy demonstrated in vaccination, including DC vaccination (90–93) and more recently, T cell receptor mimic antibodies (94, 95). Significant differences exist in the expression of LAAs relevant to CML disease progression and response, and CD34^+^ progenitor maturation in CML (81, 96), suggesting the importance of combining several antigens in future immunotherapeutic strategies. For effective activation of effector CTL responses, MHC molecules and costimulatory proteins are necessary, aberrant expression of these molecules can lead to immune evasion of cancer (26). Progenitor cells of CML patients (CD34^+^CD38^+^, CD34^+^CD38^−^, CD34^+^CD38^−^CD90^+^) actively evade host immune surveillance through cytokine-mediated downregulation of MHC-II and its master regulator class II transactivator (CIITA), a transcription factor that functions as a molecular switch for MHC-II gene regulation, and may explain why CML stem cells persist despite lifelong TKI treatment (97). CIITA plays a central role in MHC-II antigen presentation to effector CD4^+^ T cells, and thus in the stimulation of the adaptive immune response. Treatment of cells with the receptor TKI ruxolitinib (and to a lesser extent IFN-γ) enhanced the expression of MHC-II on CML stem/progenitor cells and was associated with an increase in CML cell immunogenicity (enhanced CD4^+^ T cell proliferation) in a JAK-dependent manner (97, 98). In support, clinical studies investigating the combination regimen of ruxolitinib and TKIs imatinib, dasatinib, and nilotinib in control of CML (NCT02253277, NCT01751425, and NCT01702064) are currently ongoing. The development of immunotherapeutic strategies to enhance MHC-II expression on CML stem/progenitor cells may facilitate their elimination by host immune effectors and result in greater rates of success in TFR discontinuation studies.

## The Immune System is not Permanently Compromised in CML

The introduction of TKIs has dramatically improved disease outcome in patients with CML and has replaced interferon-alpha (IFN-α) and SCT as frontline therapy (99). The therapeutic efficacy of IFN is mediated at least in part by immunological mechanisms and by the ability to cycle quiescent LSCs, responsible for disease initiation (100). The mechanism of action of TKIs, while remarkably different to that of IFN, is also accompanied by immune system re-activation, suggesting the immune system is not permanently compromised in CML (101, 102). A current limitation of immunological studies in CML is the limited investigation of immune responses longitudinally in the same patient cohort over time, for example, studies of patient samples at diagnosis, on TKI and following achievement of DMR, which may be achieved very quickly or many years later. This is further compounded by a lack of studies directly comparing the level of immune system re-activation and/or the timing of re-activation achieved with first-generation TKI imatinib compared to more potent second-generation TKIs nilotinib and dasatinib, the latter known to possess strong inhibitory activity with broader specificity against multiple other kinases including Src, Tec, and Syk family kinases, many of these being involved in innate and adaptive immune responses (103–105). Bosutinib and ponatinib are both indicated for second- or later-line treatment of CML, no studies to date appear to have explored their immunomodulatory effect on immune cell populations. We have reported maximal restoration of immune recovery in CML patients on TKI occurs only following achievement of MR^4.5^ (*BCR-ABL1* ≤0.0032%), as demonstrated by increased effector NK cell number and function and T cell immune responses, reduced numbers of PD-1^+^ CD4^+^/CD8^+^ T cells and monocytic MDSC (12). This suggests there may be a staged recovery of some immune responses in CML patients on TKI, which may be linked to depth of molecular response achieved; however, the specific causal relationship between both phenomena remains to be fully elucidated.

## The Immunomodulatory Effects of TKI

Peripheral blood DCs comprising plasmacytoid and myeloid subsets increase in number and function in CML patients following imatinib treatment (48, 106). In a murine model, DC treated with imatinib exhibited enhanced antigen-presenting cell function and restored the responsiveness of tolerant tumor-specific CD4^+^ T cells, resulting in enhanced vaccine efficacy (107). Treatment with imatinib, nilotinib, or dasatinib is associated with reduced memory B cell frequencies and significant impairment of B cell responses in CML (32). While a blunted antigen-specific immune response is observed in CML patients at diagnosis, LAA-specific CTL responses are readily detected in TKI-induced MMR and MR^4.5^ when the leukemic cell load is lower, suggesting effective restoration of anti-leukemic effector responses (12). Chen et al. (108) have also detected CTL responses in the majority of CML patients in remission on imatinib, confirming the immune system’s ability to respond to leukemia under certain conditions. It is possible the restoration of LAA-CTL responses in CML patients on TKI is closely linked to effective inhibition of aberrant PD-1 signaling, a potential consequence of diminishing antigenic stimulation in light of the significantly reduced leukemic cell load on TKI.

Dasatinib is unique in its ability to induce expansion of large granular lymphocytes (LGLs), consisting of mono- or oligo-clonal CD8^+^ T cells and NK cells, shown to correlate with better prognosis and consequently more favorable response in patients with CML (109–112). Twenty-hour pretreatment of NK cells with dasatinib followed by washout, led to dose-dependent enhancement of NK cell cytokine production, degranulation marker expression, and cytotoxicity against lymphoma and leukemia cell lines (113). Hayashi and colleagues (114) have reported dasatinib treatment markedly enhances NK cell cytotoxic function in CML patients and this is associated with an increased number of cells in the NK lineage, including CD3^−^CD56^+^ and mature CD56^+^CD57^+^ cells to levels not obtained with imatinib or nilotinib (115). CML patients in MMR and MR^4.5^ on TKI display a more mature, cytolytic CD57^+^CD62L^−^ NK cell phenotype, consistent with restoration of NK cell activating and inhibitory receptor repertoire compared to their downregulation at diagnosis (12). Comparative analysis of NK cell and T cell receptor repertoires in patients treated with imatinib frontline or nilotinib and dasatinib as first- or second-line therapy has been performed (116). The lymphocyte count and absolute number of NK cells was not significantly different between the treatment groups. Phenotypic analysis of NK cell receptors revealed dasatinib-treated patients displayed an increased expression of KIR (KIR2DL1) receptors, while imatinib-treated patients exhibited an increased expression of activating receptors (NKp30, NKp46, NKp80, and NKG2D).

**Figure 1:**
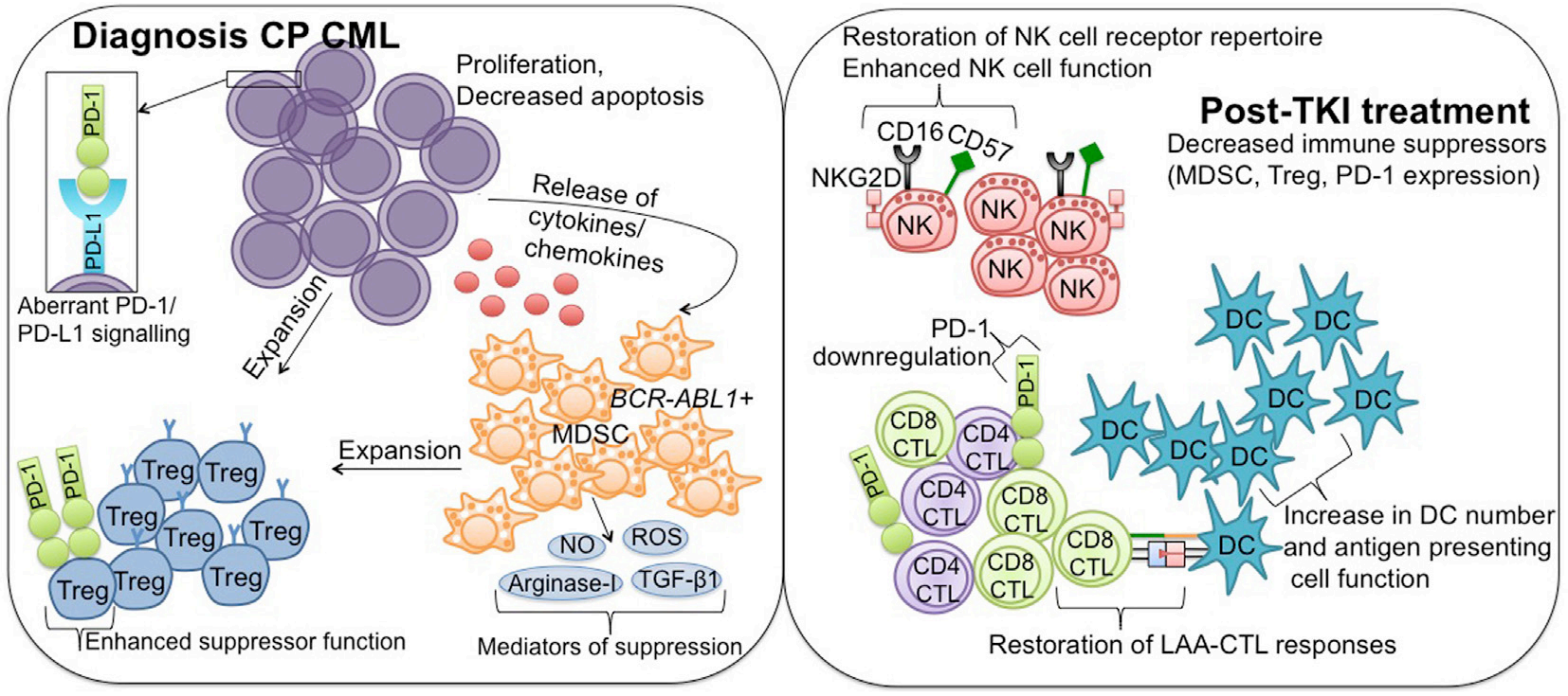
**Immune effector recovery in CML patients achieving deep molecular response on TKI therapy**. Diagnosis CP CML; suppression of the immune system in CP CML patients at diagnosis is mediated in part by hematopoietic stem cells, which acquire a proliferative/survival advantage and lose the ability to undergo apoptosis. Release of tumor-derived cytokines/chemokines drives the expansion of immune suppressor MDSC and Treg, facilitating downregulation of antitumor effector immunity. PD-L1 is upregulated on CML cells, where it interacts with the coinhibitory receptor PD-1, and contributes to protection of the malignant cells from immune destruction. MDSC originate from the malignant *BCR-ABL1* clone and mediate their suppressive activity *via* a number of mechanisms, including increased production of reactive oxygen and nitrogen species (NO, ROS), arginase-1, and TGF-β1. MDSC can induce Treg expansion, and Treg also express PD-1 to promote enhanced suppressor function. Post-TKI treatment; TKI exert immunomodulatory effects, particularly against key suppressor MDSC and Treg populations, conferring immune system re-activation and restoring effector-mediated immune surveillance. More specifically, TKI treatment leads to restoration of NK cell receptor repertoire and enhanced NK cell function, restoration of LAA-CTL responses, including downregulation of PD-1 to normal levels, and increased DC number and antigen-presenting cell function. CML, chronic myeloid leukemia; TKI, tyrosine kinase inhibitor; CP, chronic phase; MDSCs, myeloid-derived suppressor cells; Treg, regulatory T cells; PD-L1, programmed death ligand-1; PD-1, programmed death-1; NO, nitric oxide; ROS, reactive oxygen species; TGF-β1, transforming growth factor-β1; NK, natural killer; LAA, leukemia-associated antigen; CTL, cytotoxic T lymphocyte; DC, dendritic cell.

The immunostimulatory effects of dasatinib also extend to immune suppressors, as dasatinib has the potential to reduce Treg in both bone marrow and peripheral blood, skewing the balance of immune suppression toward activation and proliferation, promoting immune stimulation (115). Treg reduction has been shown to be markedly enhanced in dasatinib-treated CML patients developing LGL lymphocytosis (109, 117). Balachandran et al. (23) have found that the immune system contributes substantially to the antitumor effects of imatinib *via* the inhibition of indoleamine 2,3-dioxygenase (IDO), an important regulatory checkpoint influencing Treg expansion and activity (118). Imatinib activates CD8^+^ T cells and NK cells and leads to apoptosis of immunosuppressive Treg (23, 119). At clinically relevant doses, imatinib treatment of mice reduces Treg frequency and inhibits Treg suppressive activity and FoxP3 expression *in vivo* (120). The induction and persistence of Treg immunosuppressive function is dependent on FoxP3 (120). Zahran et al. (78) have reported Treg numbers are significantly lower in CML patients achieving DMR on imatinib therapy compared to diagnosis and patients not achieving DMR. Hayashi et al. (114) also revealed the frequency of Treg decreased in patients treated with imatinib, nilotinib, or dasatinib, with the percent decrease of Treg comparable among the three treatment groups (Figure 1).

Giallongo et al. (13) have previously shown MDSC decrease to normal levels in CML patients following imatinib therapy, and more recently, both imatinib and dasatinib have been shown to modulate the immunosuppressive CML tumor milieu, leading to decreased numbers of MDSC and associated inhibitory molecule arginase-1 expression (121). The TKI sunitinib reduces the frequency of MDSC and reverses T cell immune suppression in the peripheral blood of metastatic renal cell carcinoma patients (22).

## Contributing Immunological Factors in TFR

Several clinical trials including the landmark STIM (122, 123) and CML 8 (TWISTER) (5) trials have demonstrated that ~40% of CML patients in stable DMR successfully maintain TFR after stopping imatinib therapy. Importantly, all patients in molecular relapse (MolR) remained responsive to imatinib re-treatment, and most patients who relapsed did so within 6 months of imatinib cessation. Many other TFR stopping trials are currently ongoing, some with less stringent criteria for relapse than earlier studies, such as loss of MMR for re-starting TKI therapy and others evaluating discontinuation of second-generation TKIs (124–130). Rea and colleagues (130) provided the first report of cessation of second-generation TKI dasatinib or nilotinib (STOP 2G-TKI) in CML patients, TFR rates at 12 and 48 months were 63% (CI: 51–76%) and 55% (CI: 40–67%), respectively. Prior suboptimal TKI response or TKI resistance was the only baseline patient characteristic associated with a significantly higher incidence of MolR following cessation. The largest discontinuation study to date, the European stop TKI (EURO-SKI) trial has recently reported a molecular recurrence-free survival of 52% (CI: 48–56%) in 750 CP CML patients treated with imatinib, nilotinib, or dasatinib at 24 months, using loss of MMR as the criteria for relapse (126).

The lack of overt relapse in such patients, despite the presence of very low levels of residual disease has been attributed to immunological control of CML (131), challenging the long-held belief that SCT represents the only “curative” therapy for CML and indicating that complete elimination of residual CML stem cells is not necessary in all patients to successfully achieve TFR (132). DMR occurs in ~20% of imatinib treated patients in the first 2–3 years of therapy; however, using more potent TKIs dasatinib and nilotinib, the rates of DMR are faster and deeper, and it is possible that this will lead to more patients eligible to attempt TFR in a shorter time frame (133, 134). However, it is also likely that the optimum duration of TKI treatment before discontinuation attempt is heterogeneous and varies between patients (135). To this end, identifying predictive factors for successful discontinuation of TKI and thus, identification of CML patients who would benefit most from discontinuation remains a key issue (6).

## Role of the Immune System for Successful TFR

To date, more than 2,000 CML patients worldwide have attempted to discontinue TKI after achieving DMR in the clinical trial setting (6). More recently, several immunological sub-studies within larger discontinuation trials have attempted to identify immunological predictive factors of TFR maintenance, typically prior to TKI cessation (baseline immune markers) (6). Results from Immunostim, performed on a subset of 51 patients enrolled in the STIM trial, identified an association between elevated peripheral blood NK cells and positive clinical outcome following imatinib discontinuation (136). Accordingly, these observations provided some of the first evidence that NK cell based immune surveillance may contribute to CML control following TKI cessation. More recent molecular data (A-STIM and EURO-SKI trials) demonstrating fluctuating *BCR-ABL1* levels just below MMR without loss of MMR, and therefore without TKI resumption, has all but confirmed the importance of the role of immune surveillance for sustained TFR in CML. Immunostim did not observe any association between CD3^+^/CD4^+^/CD8^+^ T cells, the CD4/CD8 ratio, or Treg numbers in patients who relapsed compared to those who did not. Patient characteristics revealed 52.9% of the patients had received prior IFN therapy, shown in other studies to contribute to higher TFR rates (5, 137). Induction of a PR3-specific CTL response by IFN has been shown to contribute to this effect (138). Prior to the advent of TKI therapy, patients treated with IFN who discontinued treatment without relapse showed increased NK cell counts (139). IFN/imatinib induction treatment followed by a temporary IFN maintenance may enable a higher rate of treatment discontinuation in CML patients in at least MMR when stopping TKI (140). The TIGER study (NCT01657604) is currently investigating de-escalating maintenance therapy using low dose IFN as an inducer of immune surveillance following nilotinib discontinuation.

In keeping with the NK cell effector responses observed in Immunostim, a subgroup analysis of 45 patients in the EURO-SKI clinical trial has also shown that patients with higher NK cell counts at the time of TKI discontinuation are more likely to have successful TFR, linked to an increased frequency of more mature (CD57^+^) and cytotoxic (CD16^+^ and CD57^+^) NK cells (18). Prior IFN treatment showed no statistically significant correlation between relative or absolute numbers of NK cells. Similar association to successful TFR was not found with T cells or B cells or their subsets. The low affinity Fcγ receptor III (CD16) facilitates antibody-dependent cellular cytotoxicity, involved in triggering NK cell cytotoxicity (141). However, evaluation of activating NK cell surface receptors: NKG2C, NKp46, and NKG2D, which control NK cell cytotoxic responses revealed no significant difference between the patient groups. We have examined expression levels of all three natural cytotoxicity receptors NKp30, NKp44, and NKp46 and additional C-type lectin receptors NKG2A, NKG2C, NKG2D, CD161, and CD69 in Australian CML patients at time of TKI cessation and 3 and 6 months after (142). NKG2D represented the only differentially expressed receptor, decreased in MolR patients when compared to TFR at all three time points assessed. The functional degranulation response of CD3^−^CD56^dim^ NK cells following target cell stimulation does not appear to be different between TFR and MolR (18, 136, 142). In further analysis by EURO-SKI, TFR patients had more NK cells that had downregulated CD16 (CD16^−^) upon K562 stimulation, suggesting an increased activity of these cells. In addition, the TNF-α/IFN-γ cytokine secretion by these activated CD56^dim^CD16^−^ NK cells correlated with TFR success (18). A separate sub-study of 122 EURO-SKI patients has recently reported that increased CD86^+^ pDCs, which mediate immune tolerance are found in MolR patients at the time of TKI cessation, and thus low CD86^+^ pDC might be predictive of TFR (19). This study also found that higher numbers of pDCs correlated with increased PD-1-expression on PR3-specific CD8^+^ CTLs, suggesting immune exhaustion contributes to relapse risk.

The specific role and/or predictive ability of NK cells in successful dasatinib cessation is currently unclear. In the Japanese D-STOP trial, patients with DMR on TKI received dasatinib consolidation therapy for 2 years prior to cessation, and at the end of the consolidation period, there was a significant increase in the proportion of CD3^−^CD56^+^ NK cells in patients who relapsed (127). In contradistinction, the Japanese DADI trial (125) where patients received dasatinib consolidation for 1 year prior to cessation showed high NK cell (CD3^−^CD56^+^ and CD16^+^/CD56^+^) and NK cell LGL (CD56^+^CD57^+^) numbers, and low γδ T cells and Treg (CD25^+^CD127^low^) counts prior to stopping second line dasatinib treatment were associated with an increased likelihood of TFR success. Treg facilitate tumor cell immune evasion and may be a contributing factor responsible for relapse in these patients. By contrast, other studies have reported no difference in Treg or Treg naïve/memory subsets in TFR patients compared to those who relapsed (136, 142). This difference may reflect heterogeneity of distinct immune subsets, which are pre-conditioned by different TKI treatment modalities prior to cessation attempt. For example, DADI requires 1 year of dasatinib consolidation therapy prior to cessation, while Immunostim and our recently reported Australian data represent a predominantly imatinib treated patient cohort. Yoshida et al. (143) have suggested a critical role of Treg inhibition by dasatinib for the induction of NK cell effector differentiation and achievement of DMR and dasatinib has been previously shown to potently inhibit the proliferation and function of CD4^+^CD25^+^ Treg (24). Thus, it is possible the enhanced NK cell immune effector phenotype observed in patients who did not relapse in DADI, acting in concert with reduced immune suppressive Treg, reduces the likelihood of relapse in CML patients following TKI discontinuation. Further studies are needed to ascertain the mechanisms responsible for achievement of TFR in the absence of reduced Treg as it does not appear that reduced Treg numbers are critical in all TFR scenarios.

The only difference in immune suppressors observed in our Australian patient cohort was increased monocytic MDSC in MolR patients at the time of TKI discontinuation. It is possible monocytic MDSC, which play an important role in suppression of host immune responses, including effector NK responses (144) block or dampen immune surveillance, a necessary component against relapse in CML. Therapeutic strategies aimed at targeting the number and/or function of MDSC, such as promoting their differentiation into mature myeloid cells that do not have suppressive abilities, inhibition of the signaling pathways that regulate MDSC suppressive factors, or elimination of MDSC using chemotherapeutic drugs may further enhance TFR success rates (62). We have also identified reduced KIR2DL2/DL3/DS2 positive NK cells in TFR patients at 3 months and 6 months post-TKI cessation compared to MolR patients (142). In this setting, it is possible antibodies that block KIR on NK cells may enhance TFR success rates. However, inhibition of KIR2D with the monoclonal antibody IPH2101 has been shown recently to induce contraction and hyporesponsiveness of NK cells in patients with myeloma, with reductions in NK function directly correlating with loss of free KIR2D surface molecules (145). This finding may compromise antibody-based strategies designed at augmenting NK cell tumor killing *via* KIR inhibition in CML.

Killer immunoglobulin-like receptor genotypes have been reported to influence the probability of achieving molecular response in CML; however, none has yet emerged as a reliable predictor of TFR after DMR has been achieved (135). Caocci et al. (146) reported KIR polymorphisms that could be significantly associated with increased likelihood of TFR success. Interestingly, one of the haplotypes has already been reported to be significantly linked to the TKI response (KIR2DL5B) (147). However, there was no difference observed in KIR genotypes in EURO-SKI patients with TFR success compared with MolR (18).

## Concluding Remarks and Future Directions

Treatment-free remission attempts are safe and are feasible in most well-responding CP CML patients, and the impact of implementing stopping strategies as a routine part of clinical practice in CML would be significant, from both a patient-centered and medico-economic viewpoint. At present, available data show that MolR typically occurs within 6 months of TKI discontinuation, and most studies have identified the majority of clinical prognostic factors in patients prior to stopping TKI. Normalization of many immune cell populations early in the setting of TFR and MolR, at least by 3 months following the initial timepoint of TKI cessation, may be linked to the dynamic nature of the immune system as evidenced by its constant immune surveillance and the enhanced net effector immune responses and decreased PD-1 and immune suppressors observed in CML patients in DMR compared to diagnosis (12). Thus, longer follow-up of patients in all TFR studies is critically needed to determine how long patients will ultimately be able to maintain TFR (148). In this regard, immune monitoring may reveal an optimum threshold level of immune effector responses in which TKI cessation is more achievable and successful, which could prove especially informative for patients contemplating a second TKI discontinuation attempt following initial failure. Preliminary data suggest a net balance between both the effector and suppressor arms of the immune system may be important in mediating TFR success (142); however, a major goal remains to identify the most effective pathways to target to maximize an advantageous immune response and promote TFR success. Based on what is known regarding NK cell frequency in TFR, trials are underway using pharmacological manipulation, such as lenalidomide, which stimulates NK cells and enhances antitumor responses, in combination with TKI to further augment TFR success in CML (ACTRN12615001169538), or using IFN in combination with a second-generation TKI such as nilotinib (NCT02001818) to enhance immune-modulation prior to a TFR attempt. In conclusion, the identification of a robust immunological biomarker that accurately predicts which patient may stop TKI without relapse, and delineation of the precise mechanisms involved in TFR success should become a primary goal of future discontinuation studies in CML.

## Author Contributions

All authors listed have made substantial, direct, and intellectual contribution to the work and approved it for publication.

## Conflict of Interest Statement

AH declares no competing financial interests. AY has received research funding from Novartis, Bristol-Myers Squibb, and Celgene and honoraria from Novartis and Bristol-Myers Squibb.
